# Frequent Epigenetic Suppression of Tumor Suppressor Gene Glutathione Peroxidase 3 by Promoter Hypermethylation and Its Clinical Implication in Clear Cell Renal Cell Carcinoma

**DOI:** 10.3390/ijms160510636

**Published:** 2015-05-11

**Authors:** Qianling Liu, Jie Jin, Jianming Ying, Mengkui Sun, Yun Cui, Lian Zhang, Ben Xu, Yu Fan, Qian Zhang

**Affiliations:** 1 Department of Urology, Peking University First Hospital and Institute of Urology, National Research Center for Genitourinary Oncology, Beijing 100034, China; E-Mails: lqlbjmu@163.com (Q.L.); jinjie@vip.163.com (J.J.); wkdsmk@163.com (M.S.); cuiyunbjmu@163.com (Y.C.); hays0324@163.com (L.Z.); xuben123457890@163.com (B.X.); dantefanbmu@126.com (Y.F.); 2 Department of Pathology, Cancer Institute and Cancer Hospital, Peking Union Medical College (PUMC), Chinese Academy of Medical Sciences, Beijing 100021, China; E-Mail: jmying@hotmail.com

**Keywords:** *GPX3*, reactive oxygen species, methylation, tumor suppress gene, clear cell renal cell carcinoma

## Abstract

The goal of this study is to identify novel tumor suppressor genes silenced by promoter methylation in clear cell renal cell carcinoma (ccRCC) and discover new epigenetic biomarkers for early cancer detection. Reactive oxygen species (ROS) is a major cause of DNA damage that correlates with cancer initiation and progression. Glutathione peroxidase 3 (*GPX3*), the only known extracellular glycosylated enzyme of GPXs, is a major scavenger of ROS. *GPX3* has been identified as a tumor suppressor in many cancers. However, the role of *GPX3* in ccRCC remains unclear. This study aimed to investigate its epigenetic alteration in ccRCC and possible clinicopathological association. In our study, *GPX3* methylation and down-regulation were detected in 5 out of 6 ccRCC cell lines and the *GPX3* mRNA and protein expression level in ccRCC tumors was significantly lower than in adjacent non-malignant renal tissues (*p* < 0.0001). Treatment with 5-Aza-2'-deoxycytidine restored *GPX3* expression in ccRCC cells. Aberrant methylation was further detected in 77.1% (162/210) of RCC primary tumors, but only 14.6% (7/48) in adjacent non-malignant renal tissues. *GPX3* methylation status was significantly associated with higher tumor nuclear grade (*p* = 0.014). Thus, our results showing frequent *GPX3* inactivation by promoter hypermethylation in ccRCC may reveal the failure in the cellular antioxidant system in ccRCC and may be associated with renal tumorigenesis. *GPX3* tumor specific methylation may serve as a biomarker for early detection and prognosis prediction of ccRCC.

## 1. Introduction

Clear cell Renal cell carcinoma (ccRCC) is the most lethal type of urological cancer due to its occult onset and resistance to chemotherapy and radiation. Although radical nephrectomy is effective to cure local and early ccRCCs, 30% of patients develop metastases after surgery [1]. Therefore, novel, specific biomarkers for early tumor detection and anti-tumor agents are urgently required.

It is well recognized that inactivation of tumor suppressor genes (TSGs) may lead to neoplastic changes. TSGs can be inactivated by both genetic and epigenetic mechanisms, such as point mutation, LOH, and promoter hypermethylation. Recently, multiple TSGs associated with promoter hypermethylation have been identified in ccRCC, such as *VHL*, *p16*, *RASSF1A*, *SPINT2* and *HOXB13* [2,3,4,5]. Our group has also identified some TSGs silenced by promoter methylation in ccRCC, including *DLC1*, *DLEC1* and *IRF8* [6,7,8]. These findings provide a new insight to probe the molecular mechanisms of ccRCC and to seek potential diagnostic and therapeutic target for ccRCC. However, most of these known TSGs have a relatively low frequency of methylation in ccRCC. Thus, further studies are needed to identify novel and specific methylation-sensitive tumor suppressor genes in ccRCC.

Increased reactive oxygen species（ROS）levels have been found in a broad range of tumor tissues (e.g., lung, breast, esophagus and liver) [9], indicating a role for ROS as a common cause of human cancers. Under normal circumstances, ROS play a role in signal transduction. However, accumulation of excess cellular ROS has been reported to induce increased DNA mutations that have been associated with increased carcinogenesis [10].

Normal cells have integrated antioxidative systems that protect cells from ROS-induced DNA damage and cell injury. Among these systems, the glutathione peroxidase family (GPXs) is a major antioxidative enzyme family that promotes the reduction of lipid peroxides, hydrogen peroxide and organic hydroperoxide by reduced glutathione [11]. *GPX3*, located on chromosome 5q32, is the only known extracellular glycosylated enzyme of GPXs that can use thioredoxin, glutaredoxin and glutathione as electron donors, to reduce a wide range of hydroperoxides [12,13]. It is a major scavenger of ROS produced during normal metabolism or after oxidative insult. *GPX3* mRNA is expressed in a variety of normal human tissues, including kidney, liver, breast, heart, lung, brain, and gastrointestinal tract, but the majority of plasma *GPX3* is kidney-derived [13,14]. Recently, downregulation of *GPX3* by promoter hypermethylation has been reported in multiple human cancers, such as prostate, gastric, esophageal, cervical, and bladder cancer [15,16,17], suggesting that *GPX3* serves as a tumor suppressor in these cancers. However, the role and the clinical implication of *GPX3* in ccRCC remain unclear.

In this study, we examined *GPX3* expression and promoter methylation status in ccRCC cell lines and primary tumors, analyzed the relationship between its methylation and clinicopathological features in patients with RCC.

## 2. Results

### 2.1. Methylation of the GPX3 Promoter Correlates with Its Downregulation in ccRCC Cell Lines

**Figure 1:**
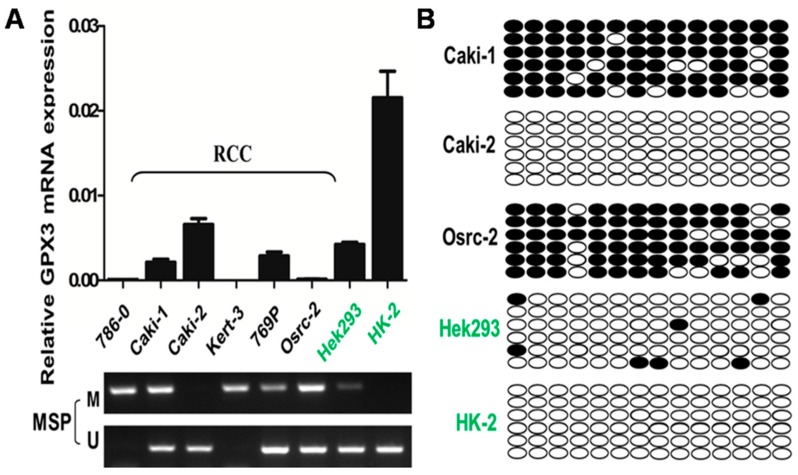
*GPX3* downregulation by promoter hypermethylation in ccRCC cell lines. (**A**) Detection of *GPX3* mRNA expression and methylation in a panel of ccRCC, Hek293, and HK-2 cell lines by real time RT-PCR and MSP. *M*, methylated*. U*, unmethylated; (**B**) Representative methylation analysis of individual CpG sites in the *GPX3* promoter by bisulfite genomic sequencing. Each row represents one bacterial clone with one circle symbolizing one CpG site. Filled ovals indicate methylated. Open ovals indicate unmethylated.

By Real-Time PCR, we detected for the first time that *GPX3* mRNA expression was downregulated in five out of six (83.3%) ccRCC cell lines compared with “normal” human embryonic kidney cell line (Hek293) and “normal” human proximal tubular cell line (HK-2) (Figure 1A). The region spanning the assumed promoter and exon 1 of *GPX3* is a typical CpG island and, thus, susceptible to epigenetic silencing. Therefore, we next explored the role of promoter methylation in silencing *GPX3* by methylation-specific PCR. Full or partial methylation was detected in five renal cancer cell lines (786-0, Caki-1, Osrc-2, Kert-3 and 769P), which showed downregulated *GPX3* expression, whereas only weak or no methylation was detected in the cell lines (Hek293 and HK-2) with *GPX3* expression (Figure 1A). To confirm MSP results, BGS was performed to identify methylation status of 14 CpG sites within the *GPX3* promoter. The bisulfite genomic sequencing results were consistent with those of methylation specific PCR in which high density methylated alleles were detected in *GPX3* downregulated 786-0, Caki-1, Kert-3 and Osrc-2 cell lines, while more unmethylated alleles were detected in Hek293 cell line and HK-2cell line (Figure 1B). These results indicated that promoter methylation was associated with the downregulation of *GPX3* in ccRCC cell lines.

### 2.2. Pharmacological Demethylation Restored GPX3 Expression in ccRCC Cells

**Figure 2:**
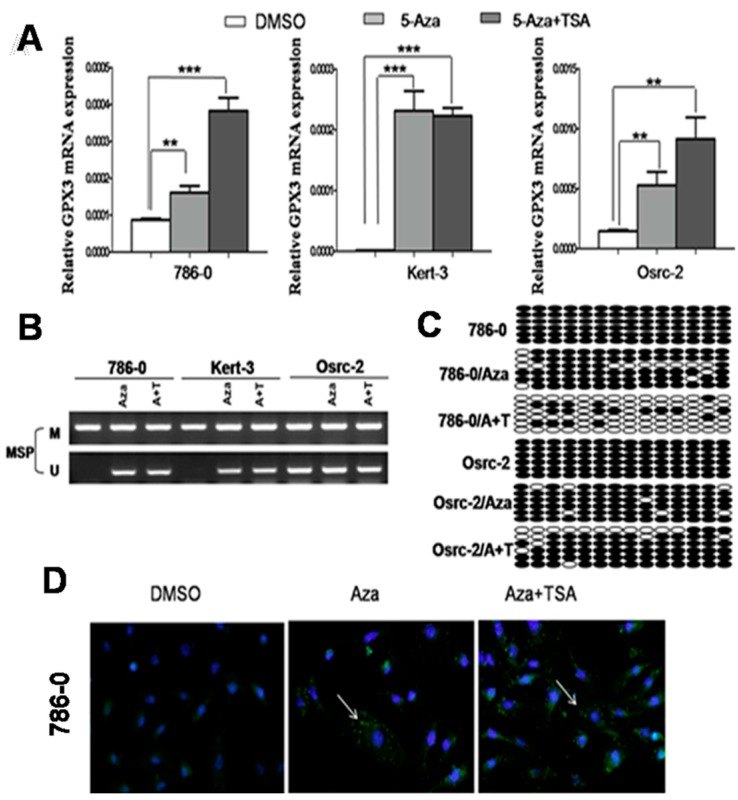
(**A**,**B**) Pharmalogic demethylation with 5-Aza alone or combined with trichostatin A (A + T) restored *GPX3* mRNA expression and induced its demethyation in ccRCC cell lines. ** *p* < 0.01; and *** *p* < 0.001; (**C**) BGS analysis of Aza and A + T treated 786-O and Osrc-2 ccRCC cells. Each row represents one bacterial clone with one circle symbolizing one CpG site. Filled ovals indicate methylated. Open ovals indicate unmethylated; (**D**) Immunofluorescence staining of *GPX3* protein in 786-O cells. Pharmalogic demethylation with 5-Aza alone or combined with trichostatin A (A + T) restored *GPX3* protein expression in 786-O cells. Green pellet in the cytoplasm represents positive staining (indicated by arrows).

To explore whether promoter methylation directly mediates *GPX3* reduction in ccRCC, three methylated cell lines that showed downregulation of *GPX3* were treated with the DNA methyltransferase inhibitor 5-Aza-2'-deoxycytidine with or without the histone deacetylase inhibitor trichostatin A. Results showed that 5-Aza treatment could restore *GPX3* mRNA expression in ccRCC cells along with a decrease in methylated alleles, and the same results were observed in A + T treated-RCC cells (Figure 2A,B). Further detailed BGS methylation analysis for 786-0 and Osrc-2 before and after Aza treatment confirmed its demethylation (Figure 2C). To check if demethylation treatment also restored *GPX3* protein level, we performed an immunofluorescence assay, using antibody against *GPX3* in 786-0 cells. As shown in Figure 2D, there was a significant increase in the *GPX3* green immunofluorescence signal after 5-Aza and 5-Aza-TSA treatments as compared to DMSO control, suggesting that pharmacological demethylation can restore the *GPX3* protein expression in 786-0 cells. These results indicated that CpG methylation of the *GPX3* promoter directly led to its suppression in ccRCC cell lines.

### 2.3. GPX3 mRNA and Protein Expression Was Frequently Downregulated or Silenced in Primary ccRCC Tumor Tissues

**Figure 3:**
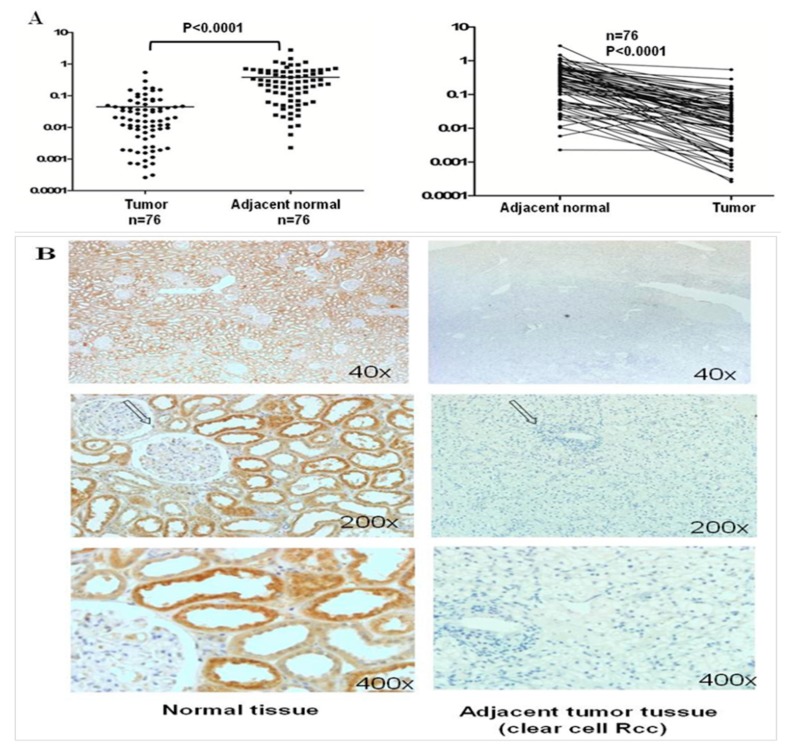

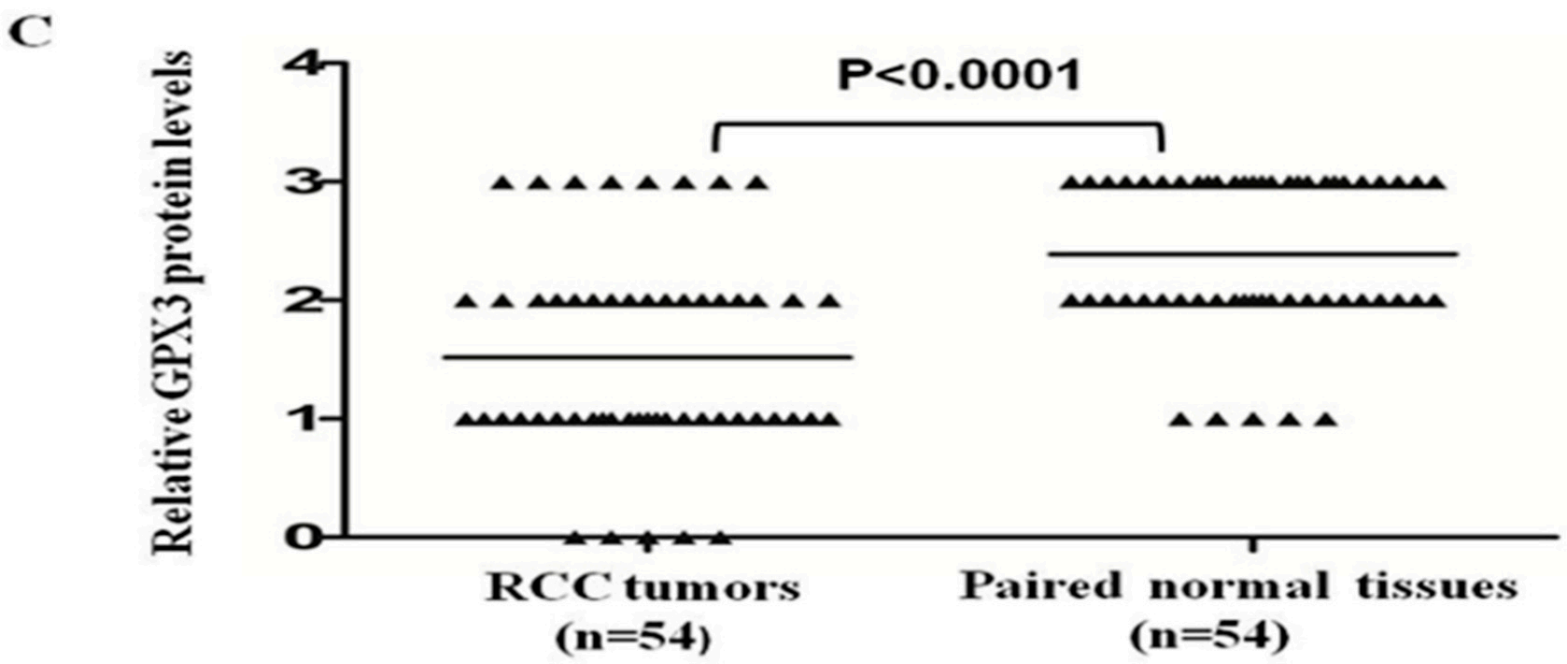
Expression pattern of *GPX3* in ccRCC*.* (**A**) Seventy-six paired ccRCC samples and adjacent non-tumor tissues were analyzed by real time RT-PCR for *GPX3* mRNA expression, which was significantly downregulated in ccRCC tumors as compared to adjacent non-tumor samples (*p* < 0.0001); (**B**) Representative immunohistochemical staining of a pair of ccRCC specimens and corresponding non-tumor tissue. In adjacent non-tumor tissues, intense immunostaining for *GPX3* was detected in a cytoplasmic distribution, whereas absent/weak immunostaining was observed in the cytoplasm of tumor tissues; The place of pictures taken in 400× was indicated by arrows; (**C**) Evaluation and statistical analysis of *GPX3* protein expression in 54 paired ccRCC samples and adjacent non-tumor tissues. *GPX3* protein expression was significantly downregulated in ccRCC samples compared to adjacent non-tumor tissues (*p* < 0.0001).

We investigated mRNA expression of *GPX3* in 76 paired ccRCC tumor tissues and their adjacent non-tumor tissues using quantitative Real-Time PCR. As shown in Figure 3A, *GPX3* was significantly downregulated in renal tumors compared with their adjacent non-tumor tissues (*p* < 0.0001). We revealed consistently low levels of *GPX3* mRNA expression in 94.74% (72 of 76) of ccRCC samples. Moreover, there was a dramatic reduction in levels of *GPX3* in 40.79% (31/76) of the cases, with a 20-fold reduction compared with the normal samples (Figure 3A). Inhibition of *GPX3* in ccRCC was further confirmed at protein level by using immunohistochemical staining. We examined *GPX3* protein expression in 54 ccRCCs and paired adjacent non-tumor tissues. In adjacent non-tumor tissues, intense immunostaining for *GPX3* was observed in a cytoplasmic distribution (Figure 3B), whereas absent/weak immunostaining was detected in the cytoplasm of tumor tissues (Figure 3B). Out of 54 ccRCC tumor tissues, *GPX3* protein was negative in five (9.3%), weakly positive in 24 (44.4%), moderately positive in 17 (31.5%), and strongly positive in eight (14.8%) (Figure 3C). In contrast, out of 54 adjacent non-tumor tissues, *GPX3* protein was negative in 0, weakly positive in five (9.3%), moderately positive in 23 (42.6%), and strongly positive in 26 (48.1%) (Figure 3C). As shown in Figure 3C, statistical analysis of the immunohistochemical results revealed that protein expression of *GPX3* in ccRCC tumor tissues was significantly lower than in adjacent non-tumor tissues (*p* < 0.0001).

### 2.4. Frequent GPX3 Promoter Methylation in Primary RCC Tumors Is Associated with Poor Prognosis

**Figure 4:**
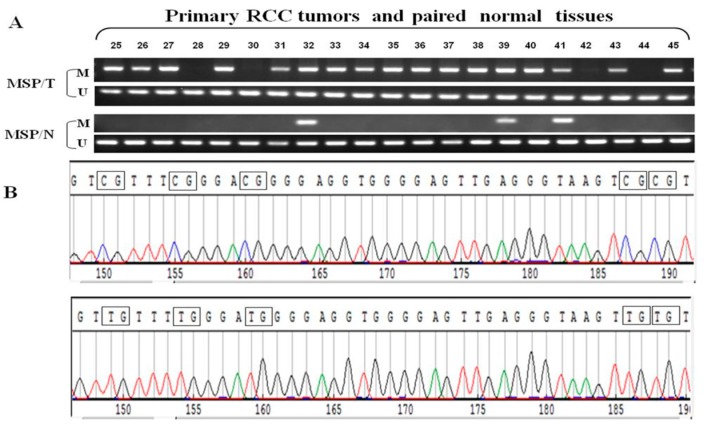
(**A**) Representative MSP results of *GPX3* methylation in RCC primary tumors (T) and paired adjacent non-tumor tissues (N). *M*, methylated; *U*, unmethylated; (**B**) Representative bisulfite genomic sequencing results of cloned BGS-PCR products. Methylated CpG sites will appear as CG during sequencing, while unmethylated CpG sites as TG.

We further examined *GPX3* methylation status in primary RCC samples and their adjacent non-tumor tissues. Results showed that *GPX3* methylation was detected in 77.1% (162/210) of RCC tumors, but only 14.6% (7/48) in adjacent non-malignant renal tissues, suggesting tumor-specific methylation of *GPX3* in RCC. Representative methylation status of *GPX3* in RCC primary tumors (T) and paired adjacent non-tumor tissues (N) are shown in Figure 4A. MSP results were confirmed by bisulfite genomic sequencing (Figure 4B). We also analyzed the correlation between *GPX3* methylation and clinicopathological features of patients with RCC. As shown in Table 1, *GPX3* methylation was significantly associated with higher tumor nuclear grade of RCC (*p* = 0.014), whereas no significant correlation was found between its methylation and gender, age, tumor location, TNM stage and histological type. In addition, our study also showed an increased percentage of GPX3 methylation in Stage 2 or higher tumors compared with Stage 1 patients (although this was not statistically significant). Collectively, these data indicate that *GPX3* methylation is a frequent event in pathogenesis of RCC and is associated with patient poor prognosis.

**Table 1:** Association between GPX3 methylation and clinicopathological features of patients with RCC.

Clinicopathological Features	Number ( *n* = 210)	GPX3 Methylation Status Methylated Unmethylated	*p* Value
**Overall**	210	162 (77.1) 48 (22.9)	
**Gender**			
M	146	112 (76.7) 34 (23.3)	0.860
F	64	50 (78.1) 14 (21.9)	(Fisher’s exact test)
**Age**			
<60 (median)	109	84 (77.1) 25 (22.9)	1.000
≥60	101	78 (77.2) 23 (22.8)	(Fisher’s exact test)
**Side**			
Rt	113	82 (72.6) 31 (27.4)	0.088
Lt	97	80 (82.5) 17 (17.5)	(Fisher’s exact test)
**TNM Classification**			
pT1a	79	56 (70.9) 23 (29.1)	0.206
pT1b	72	60 (83.3) 12 (16.7)	(chi-square test)
pT2	17	15 (83.3) 2 (16.7)	
pT3	42	32 (76.2) 10 (23.8)	
**Nuclear Grade**			
G1	48	30 (62.5) 18 (37.5)	0.014 *
G2	135	108 (80.0) 27 (20.0)	(chi-square test)
G3	27	24 (88.9) 3 (11.1)	
**Histological Type**			
Clear cell Rcc	193	150 (77.7) 43 (23.3)	0.734
papillary Rcc	9	6 (66.7) 3 (33.3)	(chi-square test)
chromophobe Rcc	8	6 (75.0) 2 (25.0)	

* Significant difference.

## 3. Discussion

Recent studies have shown increased evidences demonstrating that reactive oxygen species (ROS) are a major cause of DNA damage that correlate with a wide range of human diseases including cancer. For instance, carcinoma cells utilize ROS to stimulate their proliferation, angiogenesis, migration, and escape of apoptotic mechanism [18]. In addition, ROS promote the motility and invasion of carcinoma cells by activating protein kinase-C (PKC) and the ERK/MAPK signaling pathways, thus increasing the risk of metastasis [19,20].

The glutathione peroxidases, a family of oxidation-reduction enzymes, play central roles in balancing the signaling, immunomodulatory and detrimental effects of ROS. *GPX3*, also named plasma glutathione peroxidase, is the only known selenocysteine-containing extracellular form of GPXs and accounts for nearly all of the glutathione peroxidase activity in plasma [21]. This peculiar feature makes *GPX3* an extremely important part, not only in GPXs family, but also among cellular antioxidant system, serving as a first line of defense against ROS prior to their entry into the cell.

*GPX3* mRNA is expressed in a wide range of normal human tissues, however, downregulation of *GPX3* has been found in multiple human cancers, such as prostate, gastric, esophageal, cervical, and bladder cancer [15,16,17], suggesting its importance in human tumorigenesis. Yu, *et al.* reported that *GPX3* suppression in prostate cancer correlates with increased incidence of lymph node metastasis and poor clinical prognosis. Overexpression of *GPX3* in prostate cancer cell lines seemed to restrain tumor growth and metastasis through downregulation of c-met, a receptor tyrosine tumor transforming gene involved in a variety of cellular processes [17,22,23]. In addition, *GPX3* was found to increase apoptotic cell death by interacting directly with p53-induced gene 3 (*PIG3*) both *in vivo* and *in vitro* [24], suggesting a novel signaling pathway of *GPX3*-*PIG3* in the regulation of cell death in prostate cancer. Recently, a study in colitis-associated carcinoma reported that *GPX3*-deficient mice exhibited increased inflammation with redistribution towards pro-tumorigenic M2 macrophage subsets, increased proliferation, hyperactive WNT signaling, and increased DNA damage [25]. Knockdown of *GPX3* in the human colon cancer cell line Caco2 resulted in increased ROS production and DNA damage. These data supports a tumor suppressor role for *GPX3* via clearance of ROS and DNA damage that lead to tumor initiation and progression.

Plasma *GPX3* is mainly derived from kidney; however, in our study we found that expression of *GPX3* was significantly downregulated in primary renal tumors compared with their adjacent non-tumor tissues (*p* < 0.0001). This finding indicate that the function of *GPX3* is impaired in ccRCC, a consequence of which is likely to be an increased amount of ROS, which would induce DNA damage, driving the carcinogenic process of ccRCC.

As an alternative to genetic changes, hypermethylation of CpG rich promoter regions leading to consequent downregulation or silencing of TSGs is now recognized as an important mechanism for cancer initiation and progression. In addition to its biological relevance to malignant transformation, DNA methylation is one of the most promising biomarkers for early detection and prognosis assessments of human cancers. Recently, studies have shown a great number of aberrantly methylated TSGs in ccRCC. However, the frequencies of aberrant methylation of most classical TSGs, such as *VHL*, *p16*, *APC*, and *CDH1*, are less than 30% in ccRCC [2], indicating that these genes are probably not the major epigenetic targets for methylation silencing in ccRCC.

Downregulation of *GPX3* expression by promoter hypermethylation has been reported in 71.4% of esophageal squamous cell carcinoma, 83% of gastric cancer and 90% of prostate cancer [17,26,27], indicating its possible suppression by epigenetic change in ccRCC. To check this hypothesis, three methylated ccRCC cell lines that showed *GPX3* inactivation were treated with the hypomethylating agents. Consistent with our research expectations, *GPX3* expression was restored by demethylation treatment, suggesting that promoter methylation of *GPX3* directly led to its suppression in ccRCC cell lines. In addition, the combine treatment with TSA exerted additional stimulatory effect on GPX3 mRNA expression, suggesting that histone acetylation may also play some roles in the regulation of GPX3 in RCC*. GPX3* methylation was further detected in 77.1% (162/210) of RCC tumors, but only 14.6% (7/48) of adjacent non-malignant renal tissues, indicating that *GPX3* methylation is a tumor-specific event involved in tumorigenesis of RCC. Of note, we observed promoter hypermethylation and downregulation of *GPX3* in a small number of tumor-adjacent “normal” renal tissues. These tumor-adjacent “normal” samples, although histologically normal, they usually have some degree of changes at the molecular level. Therefore, our data suggest that promoter hypermethylation and *GPX3* suppression is possibly an early event in renal tumorigenesis. Moreover, *GPX3* hypermethylation was associated with higher nuclear grade, indicating its potential role as prognostic predictor of RCC. Similar results were noted for prostate cancer [27].

In summary, our results showing frequent *GPX3* inactivation by promoter hypermethylation in ccRCC may reveal the failure in the cellular antioxidant system, which is the first line of defense against detrimental ROS activity. In addition, this study provides new clinical implications of *GPX3* expression inactivation and promoter hypermethylation in ccRCC. First, as a tumor suppressor gene related to higher nuclear grade, *GPX3* suppression may have a negative influence on patients’ clinical outcome, and detection of *GPX3* methylation may provide prognostic information on ccRCC, especially when TSG hypermethylation can be detected in patients’ serum and urine samples, just like *RASSF1A*, tissue inhibitor of metalloproteinase-3 and *CDH1* methylation [28]. Second, *GPX3* may serve as a potential target for clinical intervention. Gene targeting therapy to enhance *GPX3* activity might be helpful in the prevention or treatment of ccRCC. *GPX3* expression is selenium dependent, since the existence of an opal stop codon in the mid section of the *GPX3* open reading frame, and thus selenium supplements in cancer treatment may augment expression of *GPX3* and could hold promise in suppressing tumor growth. Of course, further studies are urgently required to explore the functions of *GPX3* in renal tumorigenesis.

## 4. Experimental Section

### 4.1. Patients and Tissue Samples

All human primary RCCs and adjacent nonmalignant renal tissue were obtained from the urology department of Peking University First Hospital, Beijing, China, with patients’ consent according to the university policy. All cases were collected from primary surgical resection with no prior history of RCC and adjuvant therapy. Pathological diagnosis was done and confirmed at the pathology department, Institute of Urology, Peking University First Hospital. The histopathology of tumors was classified by 2002 AJCC TNM stage and Fuhrman nuclear grade.

### 4.2. Cell Culture

Six RCC cell lines (786-O, Caki-1, CaKi-2, Kert-3, 769P and Osrc-2) were obtained from Cancer Research Institute of Beijing, Beijing University, China. Hek293 (a “normal” human embryonic kidney cell line) and HK-2 (a “normal” human proximal tubular cell line), which serve as the control cell lines for RCC, were purchased from American Type Culture Collection. They were routinely cultured in RPMI 1640 or DMEM medium supplemented with 10% fetal bovine serum (GIBCO Invitrogen, Carlsbad, CA, USA) and incubated in 5% CO_2_ at 37 °C.

### 4.3. Drug Treatment

For 5-Aza-2'-deoxycytidine (Sigma, St. Louis, MO, USA) and Trichostatin A (TSA) (Sigma St. Louis, MO, USA) treatment, cell lines were grown in a 6-well plate and treated with 10 μM 5-Aza-2'-deoxycytidine for 72 h and subsequently with or without 100 nM trichostatin A for 24 h, as described previously [29]. Controlled cells were treated with an equivalent concentration of dimethyl sulfoxide.

### 4.4. Quantitative Real-Time PCR

Real-time PCR reactions were performed using GoTaq(R) qPCR Master Mix (Promega Biotech, Madison, WI, USA) according to the manufacturer’s protocol on 7500 Fast Real-Time PCR System (ABI). The primers and PCR conditions are shown in Table 2. GAPDH was used as the housekeeping gene for loading control.

**Table 2:** Primer sequences used in this study.

Gene	Primer Sequence (5'-3')	Anneal. Temp. (°C)	No. of Cycles
Real Time			
GPX3 F	CTTCCTACCCTCAAGTATGTCCG	55	45
GPX3 R	GAGGTGGGAGGACAGGAGTTCTT		
GAPDH F	GGTGGTCTCCTCTGACTTCAACA	55	45
GAPDH R	GTTGCTGTAGCCAAATTCGTTGT		
MSP			
GPX3 m1	TATGTTATTGTCGTTTCGGGAC	59	40
GPX3 m2	GTCCGTCTAAAATATCCGACG		
GPX3 U1	TTTATGTTATTGTTGTTTTGGGATG	59	40
GPX3 U2	ATCCATCTAAAATATCCAACACTCC		
BGS			
GPX3 BGS F	GGAGTTAAAAGAGGAAGGG	58	40
GPX3 BGS R	CCCAACCACCTTTCAAAC		

### 4.5. Immunohistochemistry

Fifty-four paraffin-embedded tumor tissues and paired adjacent non-tumor tissues were analyzed using immunohistochemical Staining. Briefly, the sections were deparaffinized in xylene and rehydrated by transfer through graded concentrations of ethanol to distilled water, and endogenous peroxidase activity was blocked by incubation with 3% H_2_O_2_ for 15 min at room temperature. Then, sections were submitted to antigen retrieval in a microwave (sodium citrate buffer, pH 6.0) for 10 min, naturally refrigerated to room temperature. Blocking was performed with 10% goat serum for 30 min at room temperature. All sections were incubated with mouse anti-*GPX3* monoclonal antibody (Clone 23B1, 1:100, Abcam, Cambridge, MA, USA), overnight at 4 °C, and then incubated with goat anti-mouse secondary antibody for 30 min at room temperature. After rinsing three times in PBS for 5 min each, the sections were incubated with DAB for 2 min, counterstained with hematoxylin for 3 min, dehydrated with gradient alcohol and transparentized with dimethylbenzene. Immunohistochemical expression of *GPX3* was examined via light microscopy. Tissues were graded on the following scale: 0, negative; 1, weakly positive; 2, moderately positive; 3, strongly positive.

### 4.6. Immunofluorescence

To check *GPX3* protein expression after demethylation treatment, we performed immunofluorescence staining against *GPX3* in 786-0. Cells were fixed with 4% paraformaldehyde for 15 min, followed by permeabilization with 0.1% Triton X-100 for 5 min. Then, after washing with PBS, the cells were blocked with goat serum for 30 min. For immunofluorescence, Cells were incubated with primary antibody against *GPX3* overnight at 4 °C followed by secondary goat anti-mouse antibody conjugated with FITC (1:200 dilution; Invitrogen, Carlsbad, CA, USA) at room temperature for 45 min. The nuclei were counterstained with DAPI (1 μg/mL; Roche, Indianapolis, IN, USA) and viewed under a fluorescence microscope.

### 4.7. Bisulfite Treatment and Methylation-Specific PCR

The bisulfite modification of purified genomic DNA was performed using an EpiTect Bisulfite Kit (Qiagen 59104, Hilden, Germany) following the manufacturer’s instructions. Table 2 lists MSP primers and PCR conditions. MSP primers were examined previously for not amplifying any unbisulfited DNA and MSP products of several ccRCC cell lines were confirmed by direct sequencing, indicating that our MSP system was specific.

### 4.8. Bisulfite Genomic Sequencing

Bisulfite-treated DNA was amplified with primers specific for a fragment of the *GPX3* promoter CpG islands that contained 14 CpG sites. Table 2 lists BGS primers and PCR conditions. The PCR products were subcloned into the pEasy-T5 vector (Transgene, Beijing, China) and 5–8 colonies were randomly chosen and sequenced.

### 4.9. Statistical Analysis

Statistical analyses were tested using the 2-tailed *t*-test, Fisher exact test or chi-square test with *p* < 0.05 considered significant.

## 5. Conclusions

To our knowledge, we report for the first time that *GPX3* is frequently downregulated in renal tumors compared with their adjacent non-tumor tissues, indicating its role as a tumor suppressor. Aberrant methylation was an important reason for the suppression of *GPX3* and may be associated with renal tumorigenesis. *GPX3* tumor specific methylation may serve as a potential biomarker for early detection and prognosis prediction of RCC, especially when *GPX3* hypermethylation can be detected in patients’ serum and urine samples.

## Abbreviations

Aza: 5-Aza-2'-deoxycytidine
BGS: bisulfite genomic sequencing
GPX3: glutathione peroxidase 3
LOH: loss of heterozygosity
MSP: methylation- specific PCR
PCR: polymerase chain reaction
RCC: renal cell carcinoma
ROS: reactive oxygen species
TSA: trichostatin A
TSG: tumor suppressor gene
